# Diagnostic Performance of ChatGPT-4o in Detecting Hip Fractures on Pelvic X-rays

**DOI:** 10.7759/cureus.86654

**Published:** 2025-06-24

**Authors:** Turgut Emre Erdem, Alper Kirilmaz, Ahmet Fevzi Kekec

**Affiliations:** 1 Orthopedics and Traumatology, HG Hospital, Kahramanmaras, TUR; 2 Orthopedics and Trauma, Konya City Hospital, Konya, TUR; 3 Orthopedics and Traumatology, Necmettin Erbakan University, Konya, TUR

**Keywords:** artificial intelligence, chatgpt-4o diagnostic performance, fracture detection, hip fracture, pelvic x-ray

## Abstract

Introduction: Hip fractures are a major orthopedic problem, especially in the elderly population. Hip fractures are usually diagnosed by clinical evaluation and imaging, especially X-rays. In recent years, new approaches to fracture detection have emerged with the use of artificial intelligence (AI) and deep learning techniques in medical imaging. In this study, we aimed to evaluate the diagnostic performance of ChatGPT-4o, an artificial intelligence model, in diagnosing hip fractures.

Methodology: A total of 200 anteroposterior pelvic X-ray images were retrospectively analyzed. Half of the images belonged to patients with surgically confirmed hip fractures, including both displaced and non-displaced types, while the other half represented patients with soft tissue trauma and no fractures. Each image was evaluated by ChatGPT-4o through a standardized prompt, and its predictions (fracture vs. no fracture) were compared against the gold standard diagnoses. Diagnostic performance metrics such as sensitivity, specificity, accuracy, positive predictive value (PPV), negative predictive value (NPV), receiver operating characteristic (ROC) curve, Cohen’s kappa, and F1 score were calculated.

Results: ChatGPT-4o demonstrated an overall accuracy of 82.5% in detecting hip fractures on pelvic radiographs, with a sensitivity of 78.0% and specificity of 87.0%. PPVs and NPVs were 85.7% and 79.8%, respectively. The area under the ROC curve (AUC) was 0.825, indicating good discriminative performance. Among 22 false-negative cases, 68.2% were non-displaced fractures, suggesting the model had greater difficulty identifying subtle radiographic findings. Cohen’s kappa coefficient was 0.65, showing substantial agreement with actual diagnoses. Chi-square analysis revealed a strong correlation (χ² = 82.59, *P* < 0.001), while McNemar’s test (*P* = 0.176) showed no significant asymmetry in error distribution.

Conclusions: ChatGPT-4o shows promising accuracy in identifying hip fractures on pelvic X-rays, especially when fractures are displaced. However, its sensitivity drops significantly for non-displaced fractures, leading to many false negatives. This highlights the need for caution when interpreting negative AI results, particularly when clinical suspicion remains high. While not a replacement for expert assessment, ChatGPT-4o may assist in settings with limited specialist access.

## Introduction

Hip fractures are a major orthopedic problem, especially in the elderly osteoporotic population, and are associated with significant morbidity and mortality. According to current estimates, approximately 300,000 hip fractures occur annually in the United States alone; this number is expected to reach 500,000 in 2040 [1,2]. The one-year mortality rate in hip fractures has been reported to be as high as 23%-34% in the literature [2]. Therefore, early diagnosis and treatment of these fractures are of critical importance in terms of patient survival and quality of life.

Hip fractures are usually diagnosed through clinical evaluation and imaging. In particular, X-ray radiography is the first step in the diagnostic process. Although the diagnosis can be made with radiographs in most cases, the fracture may not be evident in some cases. If the suspicion is high but the X-ray is normal, advanced tests such as computed tomography (CT) imaging and magnetic resonance imaging (MRI) can be used to confirm the diagnosis [3,4].

In recent years, new approaches to fracture detection have emerged with the use of artificial intelligence (AI) and deep learning techniques in medical imaging. In the literature, it has been shown that deep learning models, especially convolutional neural networks, can detect fractures in radiographs with high accuracy [5].

On the other hand, large language models (LLMs)-based chatbots, such as ChatGPT-4o, have also started to gain traction in the medical field. ChatGPT-4o (GPT-3.5 and GPT-4) is an advanced language model trained on a huge corpus of text, capable of producing human-like text [6].

Due to its extensive training on medical literature, it has demonstrated the theoretical ability to answer medical questions, conduct case discussions, and even answer exam questions. ChatGPT-4o has been reported to perform at a level that can pass the United States Medical Specialty Examinations (USMLE) [7]. This suggests that LLMs could be a potentially impactful development in healthcare. In one study, it was reported that ChatGPT-4o showed significant improvement in clinical case questions compared to the previous version, achieved 87% accuracy in USMLE step 2 questions, and was able to list the correct diagnosis in the top three possible diagnoses 74.6% of the time in real clinical case scenarios [8]. Another study has shown that chatbots using ChatGPT-4o technology can generate AO codes from radiologic reports. They noted that the chatbot measured significantly faster than humans but was less reliable in generating AO codes [9].

There are very few studies in the literature that show ChatGPT-4o is helpful in the diagnosis of fractures. Previously, radiologic evaluation was performed on fractures around the knee, and it was reported that ChatGPT-4o could diagnose tibia plateau fractures similar to emergency physicians and radiologists [10]. In another study, the diagnostic and treatment planning performance of ChatGPT-4o, DeepSeek-V3, and Gemini 1.5 models for hand fractures was evaluated in a comparative study. A total of 58 anonymized hand fracture cases were presented to each model, and their clinical recommendations were compared with the decisions of experienced hand surgeons. Among the models, ChatGPT-4o demonstrated the highest performance with an accuracy of 98.28% [11].

While many radiology-focused AI models have demonstrated excellent diagnostic performance [5], the aim of this study was to evaluate whether a more widely accessible and general-purpose language model, such as ChatGPT, commonly encountered in everyday use, could provide meaningful diagnostic assistance when applied to radiographic data.

## Materials and methods

**Figure 1:**
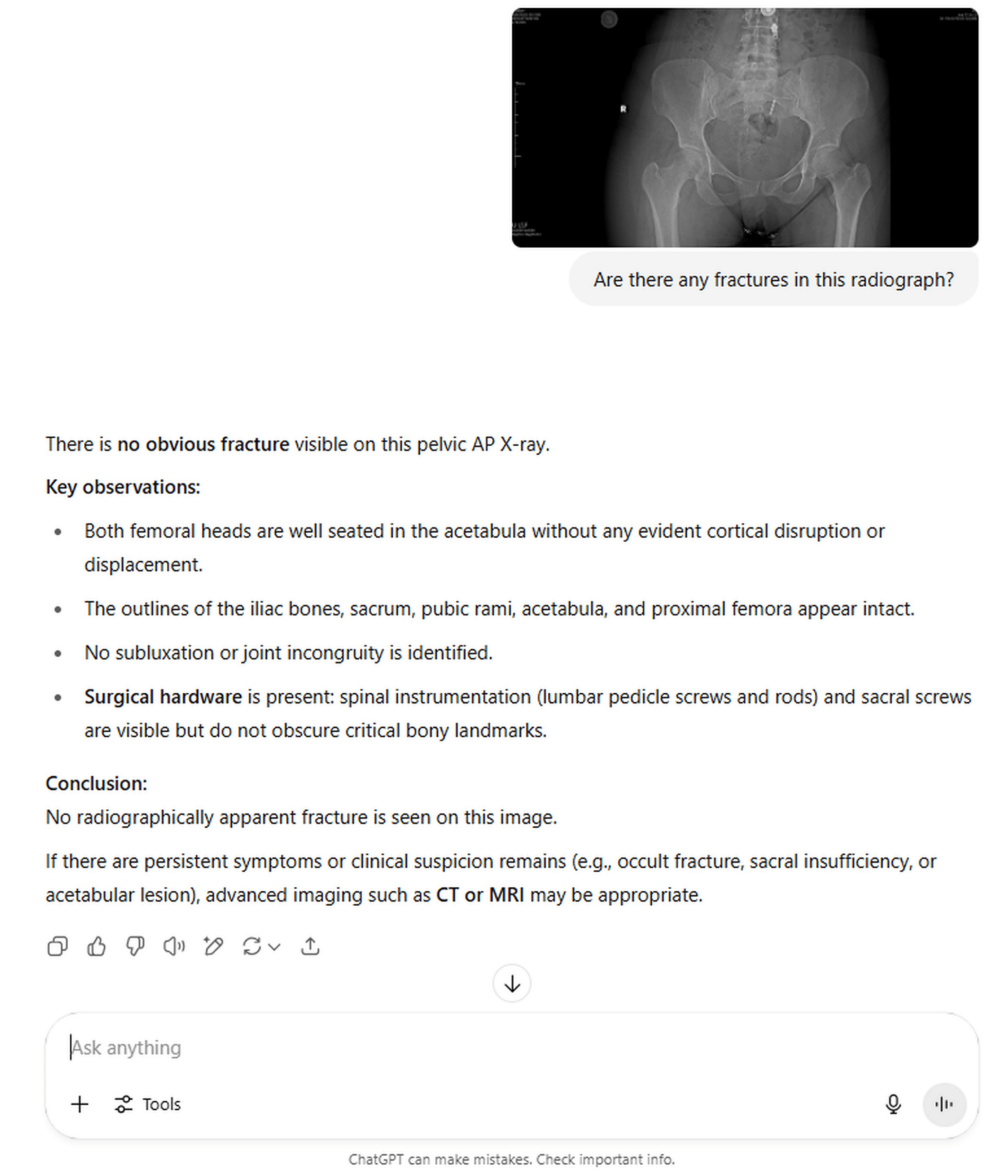
ChatGPT-4o evaluation of an X-ray image with no observed fracture.

**Figure 2:**
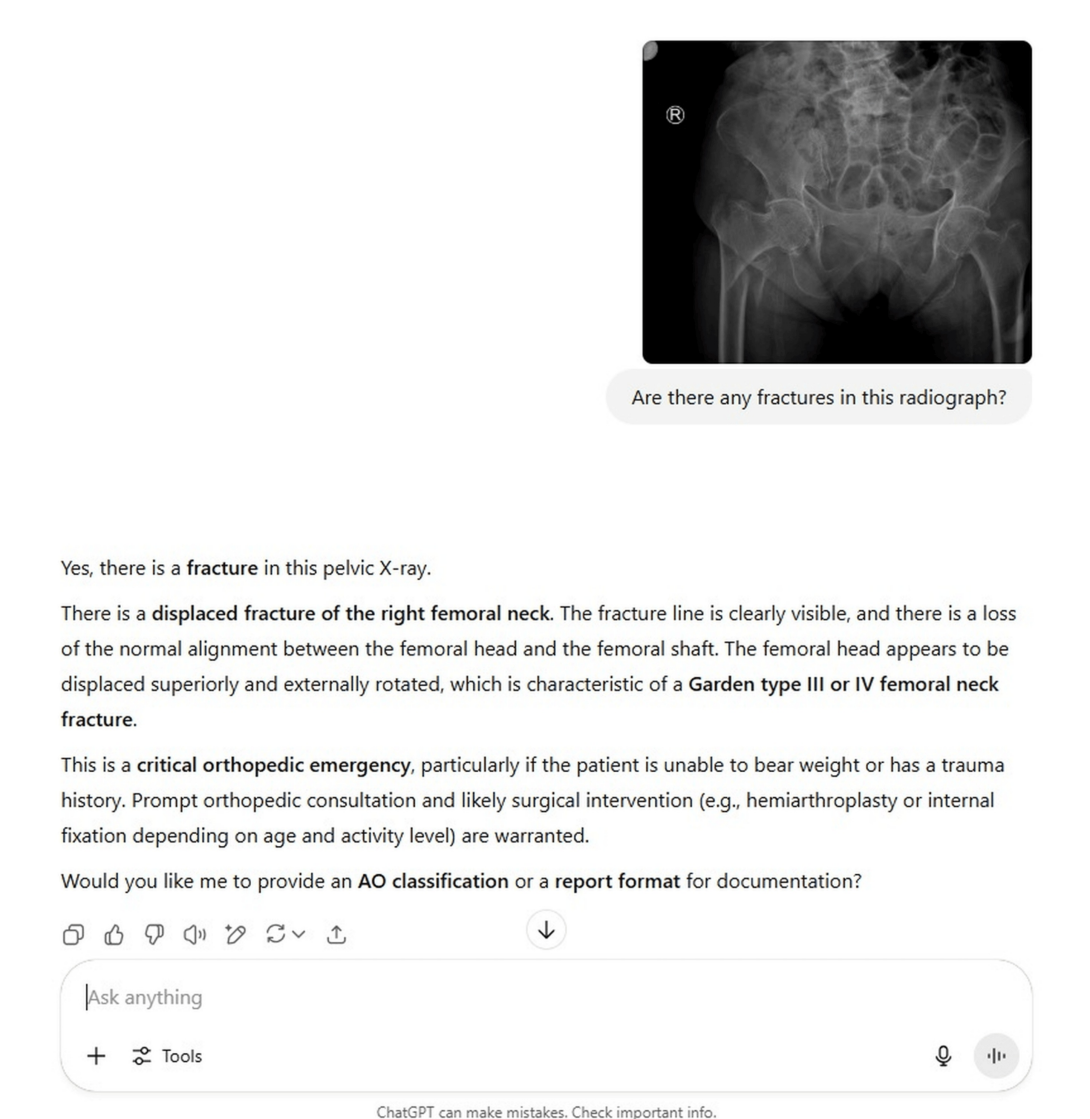
ChatGPT-4o evaluation of an X-ray image showing a hip fracture.

This single-center, retrospective study was conducted at the Department of Orthopedics and Traumatology at Necmettin Erbakan University between January 2022 and January 2025. A total of 200 anteroposterior (AP) pelvic radiographs were included in the study. For all patients diagnosed with a hip fracture, CT images were available. Fracture diagnoses were established independently by two orthopedic and trauma surgeons in two separate sessions, using both CT scans and AP pelvic radiographs, in a blinded manner. Patients with periprosthetic fractures or pelvic ring fractures accompanying proximal femur fractures were excluded from the study to ensure diagnostic homogeneity. Among these, 100 radiographs were obtained from patients who underwent surgery for a hip fracture diagnosis; of these, 32% were non-displaced fractures and 68% were displaced fractures. The remaining 100 radiographs belonged to patients diagnosed with soft tissue trauma without any hip fracture. All images were uploaded to the ChatGPT-4o system in JPEG format and a randomized order. For every image, a distinct ChatGPT query tab was used and asked, “Are there any fractures in this radiograph?” (Figures 1-2). The “fracture” rating given by ChatGPT-4o was recorded. The actual fracture status was defined as a binary variable (fracture present or absent), as to whether the patient had a fracture in the corresponding femoral region. The ChatGPT-4o assessment was also noted for each case in the same way as “fracture present” or “fracture absent.”

Sensitivity and specificity, ROC curve and AUC analyses, chi-square compliance test, McNemar test, Cohen's kappa analysis, and F1-score calculations were performed to measure the relationship between actual fracture status and ChatGPT-4o assessment and the discriminative power of the model. All statistical analyses were conducted using a two-tailed significance threshold of *P* < 0.05. Analyses were performed in the Python environment. The statsmodels library was used for parametric tests and analyzing categorical data, while scikit-learn was used for ROC analysis and evaluating performance metrics. Results were summarized in tables and figures.

## Results

One hundred cases had a true hip fracture, while the remaining 100 cases had no fracture (gold standard). ChatGPT-4o classified 91 of these cases as “fracture present” and 109 as “fracture absent.” The relationship between ChatGPT-4o predictions and actual diagnoses is presented in Table 1, and model performance measures are presented in the related text. Additionally, the F1-score, which reflects the harmonic mean of precision and recall, was calculated as 0.817, indicating balanced and robust classification performance.

**Table 1:** Association between real diagnoses and ChatGPT-4o predictions.

	ChatGPT: Fracture	ChatGPT: No fracture	Row total
Fracture present	78 (True positive)	22 (False negative)	100
Fracture absent	13 (False positive)	87 (True negative)	100
Column total	91	109	200
Test	Test statistics	*P*-value	
Pearson chi-square test	χ² = 82.59	<0.001	
McNemar’s test	χ² = 1.829	0.176	

According to Table 1, ChatGPT-4o correctly identified 78 out of 100 cases with fracture as “fracture present” and missed 22 cases with fracture as “no fracture.” On the other hand, in 87 out of 100 cases without fracture, ChatGPT-4o correctly identified “no fracture” and incorrectly identified “fracture” in 13 intact cases. The key performance metrics calculated based on these results are as follows (Table 2). Additionally, the F1-score, reflecting the harmonic mean of precision and recall, was calculated as 0.817, indicating balanced and robust classification performance.

**Table 2:** Performance metrics of ChatGPT-4o in diagnosing hip fracture. AUC, area under the curve; ROC, receiver operating characteristic

Metric	Value (%)	Description
Sensitivity	78.0	78 out of 100 fracture cases were correctly identified
Specificity	87.0	87 out of 100 non-fracture cases were correctly identified
Positive predictive value (PPV)	85.7	78 of 91 predicted fractures were true fractures
Negative predictive value (NPV)	79.8	87 of 109 predicted non-fractures were truly intact
Overall accuracy	82.5	165 out of 200 cases correctly classified
ROC AUC		AUC = 0.825, indicating good discriminative ability

ROC curve and AUC

**Figure 3:**
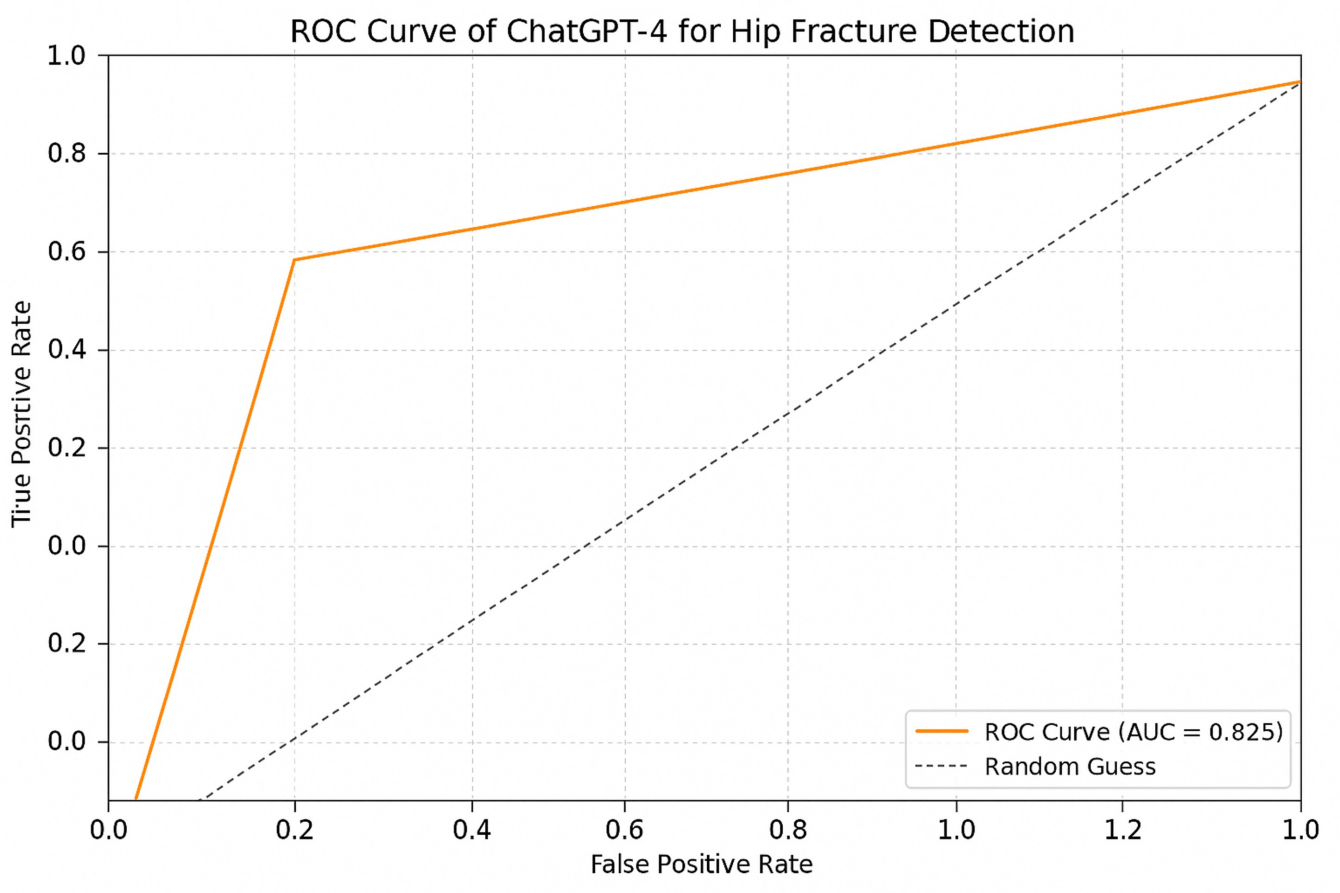
ROC curve of ChatGPT-4o for the diagnosis of hip fracture. Area under the curve (AUC) = 0.825. ROC, receiver operating characteristic

The ROC curve summarizing ChatGPT-4o’s diagnostic performance is shown in Figure 3 (AUC = 0.825).

Kappa concordance analysis

The agreement between ChatGPT-4o’s decisions and the actual diagnoses was assessed by Cohen’s kappa coefficient, which yielded a value of 0.65, indicating substantial agreement according to the Landis and Koch classification (0.61-0.80 = substantial).

Chi-square and McNemar tests

The association between ChatGPT-4o’s predictions and actual fracture status was highly significant (Pearson chi-square χ²(1) = 82.59, *P* < 0.001), indicating a strong correlation. The McNemar test, used to evaluate the symmetry of error types (false positives vs. false negatives), showed no statistically significant difference (χ² = 1.829, *P* = 0.176). This suggests that ChatGPT-4o's errors were balanced, with no evident bias toward either positive or negative classifications (i.e., a tendency to consistently predict "fracture"). In other words, the model did not exhibit a systematic error bias in either direction (Table 1).

## Discussion

This study demonstrates that ChatGPT-4o, a general-purpose AI language model, can perform remarkably well in a specific clinical task: the diagnosis of hip fracture. The accuracy of 82.5%, the sensitivity of 78.0%, and the specificity of 87.0%. This result reinforces the idea of using ChatGPT-4o as a support tool in informed decision-making processes that do not rely directly on image analysis.

A review of 14 studies using deep learning algorithms (most commonly GoogLeNet and DenseNet) reported that these models achieved hip fracture detection accuracy ranging from 79.3% to 98%, with AUC values between 0.90 and 0.99 [5]. Bae et al. used a modified spatial attention module (CBAM++) and a ResNet18 architectural model and reported an accuracy rate of 97.1% [12]. Beyaz et al. reported the lowest accuracy rate of 79.3% using a CNN containing GA blocks architectural model, and Yamada et al. reported the highest accuracy rate of 98% using the Xception architectural model [13,14]. In our study using the ChatGPT-4o model, we achieved 82.5% accuracy and an AUC of 0.825 in detecting hip fractures, which is comparable to the average performance of deep learning models.

In a previous study, various clinical cases were analyzed between general medical specialty trained specialists and residents and ChatGPT-4o, and it was shown that ChatGPT-4o alone significantly outperformed the performance of experienced physicians in complex medical cases. However, they reported that the use of LLMs by physicians did not improve diagnostic reasoning in challenging clinical cases compared to traditional support (Google, etc.) search robots [15]. In our study, we found that ChatGPT-4o alone correctly interpreted X-ray radiographs of hip fractures with moderate specificity and sensitivity. However, we did not compare the power of the AI model and experienced physicians in interpreting radiographs for hip fractures. Also, in our study, the AI model evaluated only X-ray radiographs, and we did not include cases in which diagnosis was not possible on X-ray radiographs and advanced diagnostic methods such as CT or MRI were required. This is one of the limitations of our study.

Our findings demonstrate the potential for models such as ChatGPT-4o to be used as a helpful tool in clinical settings. For example, in a case where a general practitioner in the emergency department clinically suspects a hip fracture but has difficulty interpreting the radiograph, they might ask ChatGPT-4o, "Are there any fractures in this image?" While this approach shows promise, it cannot replace definitive diagnostic tools. However, decision support can be provided, especially in settings where access to specialists and advanced diagnostic modalities such as CT and MRI is limited. Indeed, some experts suggest that advanced AI systems such as ChatGPT-4o may serve as a kind of 'doctor’s assistant' or second-opinion mechanism [16].

The points emphasized in the literature on the reliability and limitations of ChatGPT-4o are consistent. The model may alleviate the workload of clinicians by standing out with its ability to quickly access and summarize medical information [17].

In this study, ChatGPT-4o demonstrated a moderate level of diagnostic performance, given the model’s general-purpose architecture and lack of radiologic specialization. This performance, while promising, appears to surpass the results of Hiredesai et al., who investigated the diagnostic ability of ChatGPT-4o for six common upper extremity bony pathologies, including distal radius and humerus fractures, using multiple imaging modalities (X-ray, CT, MRI)​. In their study, ChatGPT-4o provided a diagnosis in only 52% of the cases, with diagnostic accuracy varying widely between 0% and 55%, depending on the pathology and imaging modality. Notably, humerus fractures, arguably the most radiographically distinct among the included conditions, had the highest diagnostic accuracy (55%), while pathologies such as carpometacarpal arthritis and scaphoid nonunion had 0% accuracy. These results underscore the variability in ChatGPT-4o's performance across anatomical regions and imaging complexities. Unlike their multi-modal and multi-pathology setup, our study focused on a binary classification (fracture vs. no fracture) within a single anatomical site using standardized AP pelvic radiographs, which likely contributed to improved model performance. Nevertheless, both studies highlight the potential of ChatGPT-4o as a diagnostic support tool, while also emphasizing the current limitations in complex or subtle musculoskeletal imaging interpretations [18].

In a recent study by Zhu et al., ChatGPT-4o demonstrated strong binary classification performance in detecting the presence or absence of knee osteoarthritis but showed limited accuracy when tasked with more complex grading, such as the Kellgren-Lawrence scale. This finding suggests that while ChatGPT-4o excels at identifying the presence of a pathology, its performance may decline when more nuanced or tiered clinical judgments are required [19]. Similarly, in our study, ChatGPT-4o showed a marked decline in performance when evaluating non-displaced hip fractures, which require more nuanced radiographic interpretation.

One study compared ChatGPT-4o with orthopedic surgeons in the ability to diagnose Arbeitsgemeinschaft für Osteosynthesefragen/Orthopedic Trauma Association (AO/OTA) classification A1 (stable) and A1 (unstable) fractures in femoral pertrochanteric fractures, and reported that the AI was able to perform the classification similarly to surgeons. But further improvements are needed for optimal performance in radiologic diagnostics [20]. In our study, we asked ChatGPT to diagnose fractures rather than perform AO/OTA classification, and we observed that it was able to diagnose hip fractures at a moderate rate. However, we found that the radiologic diagnostic skills were not perfect.

Several important limitations of this study should also be considered. First, the relatively small sample size may limit the generalizability of the findings. In addition, potential biases inherent to LLMs may influence the model's decision-making process, which should be carefully evaluated in clinical contexts. Furthermore, the phrasing of the prompt - being brief and direct - plays a significant role in shaping the model’s responses. Each of these factors must be taken into account when interpreting model performance, and future studies should systematically investigate the impact of prompt design and model behavior using larger datasets.

## Conclusions

The model’s lower sensitivity in non-displaced fractures (68.2% of false negatives) highlights the need for caution when interpreting negative AI results in clinically suspicious cases.

Despite lacking task-specific training, ChatGPT-4o showed moderate accuracy in detecting hip fractures and may assist in low-resource settings.

Future versions may offer improved performance, and this study can serve as a reference point for upcoming research on LLMs in radiology.
